# The roles of extracellular vesicles in gastric cancer development, microenvironment, anti-cancer drug resistance, and therapy

**DOI:** 10.1186/s12943-019-0967-5

**Published:** 2019-03-30

**Authors:** Tingting Huang, Chunli Song, Lei Zheng, Ligang Xia, Yang Li, Yiwen Zhou

**Affiliations:** 1 Department of Clinical Laboratory Medicine, Shenzhen Hospital, Southern Medical University, No. 1333, Xinhu Road, Baoan District, Shenzhen, 518020 Guangdong People’s Republic of China; 2 Department of Laboratory Medicine, Nanfang Hospital, Southern Medical University, No.1838 North Guangzhou Avenue, Guangzhou, 510515 Guangdong People’s Republic of China; 3 Department of Gastrointestinal Surgery, Second Clinical Medical College of Jinan University, Shenzhen People’s Hospital, Shenzhen, 518020 Guangdong People’s Republic of China

**Keywords:** Gastric cancer, Extracellular vesicles, Exosomes, Tumor microenvironment, Drug resistance

## Abstract

Gastric cancer (GC) is one of the leading causes of cancer-related death in both men and women due to delayed diagnosis and high metastatic frequency. Extracellular vesicles (EVs) are membrane-bound nanovesicles which are released by cells into body fluids such as plasma, saliva, breast milk, cerebrospinal fluid, semen, urine, lymphatic fluid, amniotic fluid, sputum and synovial fluid. EVs deliver almost all types of biomolecules such as proteins, nucleic acids, metabolites, and even pharmacological compounds. These bioactive molecules can be delivered to recipient cells to influence their biological properties, modify surrounding microenvironment and distant targets. The extensive exploration of EVs enhances our comprehension of GC biology referring to tumor growth, metastasis, immune response and evasion, chemoresistance and treatment. In this review, we will sum up the effects of GC-derived EVs to the tumor microenvironment. Moreover, we will also summarize the function of microenvironment-derived EVs in GC and discuss how the bidirectional communication between tumor and microenvironment affect GC growth, metastatic behavior, immune response, and drug resistance. At last, we prospect the clinical application viewpoint of EVs in GC.

## Background

Gastric cancer (GC) is one of the most common and deadliest types of cancer worldwide. It is the 3rd leading cause of cancer-related death in men and 5th in women [1]. *Helicobacter pylori* (*H. pylori*) infection, Epstein–Barr virus (EBV) infection, chronic gastritis, the diet, and some genetic alterations are risk factors in the development of GC. Despite advances in diagnostic modalities and the development of molecular-targeted drugs in the clinic, the 5-year survival rate of GC is rather low. Recently, four molecular classifications on the basis of the Cancer Genome Atlas (TCGA) research network has been identified, which are EBV-associated tumors, microsatellite unstable tumors (MSI), genomically stable tumors (GS), and tumors with chromosomal instability (CIN) [2].

**Fig. 1:**
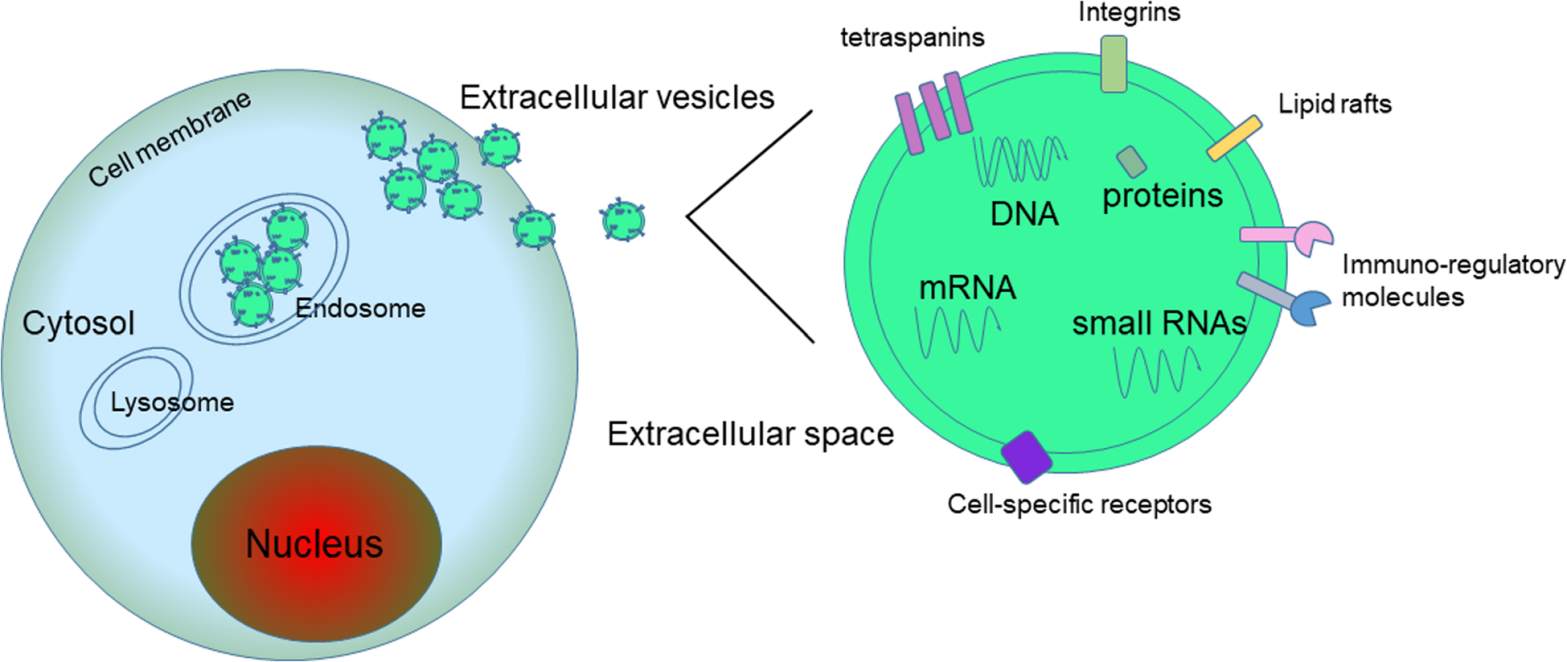
Release of EVs and its contents. Primarily, the EVs are originally derived from lysosomes and late endosomes. Then, they can be released into the extracellular environment. The contents of EVs, which contain DNAs, mRNAs, small RNAs, and proteins can be transferred from the original cell to their target cells in local microenvironment or at distant site that can possibly give rise to intercellular communication networks. Abbreviations: EVs, extracellular vesicles

Extracellular vesicles (EVs) are secreted by nearly almost cell types and released to the extracellular space. Traditionally, EVs are subgrouped into three classes according to their size: exosomes (30–100 nm in diameter), microvesicles (MVs, 100–1000 nm in diameter), and apoptotic bodies (1000–5000 nm in diameter). Exosomes are small membrane nanovesicles which constituted through the intraluminal budding of the late endosomal membrane and are secreted from the plasma membrane. MVs are efflux directly from the plasma membrane through ectocytosis and apoptotic bodies are occurred through plasma membrane“blebbing” during programmed cell death [3–6]. In both physiological and pathological conditions, EVs are released from cell membranes throughout the body including a wide range of DNAs, mRNAs, multiple proteins, microRNAs (miRNA), long non-coding RNAs (LncRNAs), circular RNAs, and metabolites (Fig. 1). These bioactive substances make interactions among tumor cells, surrounding tumor microenvironment, and distant organs and tissues. The tumor microenvironment contains complex components, such as stromal cells, endothelial cells, immune cells. Therefore, EVs, especially exosomes, are well known with their intercellular communications during tumor progression. Moreover, accumulating evidence suggests that EVs can function as intercellular transport systems according to their contents. The analysis of the contents can help us unveil the function of EVs in cancer, which might be used to identify new biomarkers in cancer diagnosis and therapy. Although there is much unknown and many inconsistent findings in the functions of EVs in cancer development, EVs have enormous potential to be used in clinical practice in the immediate future as the field rapidly expands. In this review we will describe the key findings on how tumor-derived EVs regulated cancer cell development, metastasis, immune response, drug resistance or communicated with microenvironment in GC. Moreover, we will summarize the multifaceted roles of tumor microenvironment-derived EVs in GC. The potential utility of exosomes as noninvasive biomarkers and in therapy for GC will also be discussed.

## Roles of tumor-derived EVs in GC

### Characterization of tumor-derived EVs in GC

EV is a general term to describe virtually any type of membrane particle released by cells. EVs play a critical role in communications between tumor cells themselves and tumor cells with the microenvironment. In cancer patients, EVs located in body fluid and tumor microenvironment to effect cancer progression. They could directly interact with autologous cancer cells within 2 h and then were internalized by them at 24 h as messengers transfer between GC cells to enhance tumor growth have been proved [7]. The cancer-derived EVs’ signature distinguishes them from normal cell secreted EVs. The MVs size within the range of 10–800 nm in patients, while in control MVs showed within the range of 10–400 nm. Atomic force microscopy confirmed MVs size heterogeneity with implication that larger objects represented aggregates of smaller microparticles. In patients’ MVs, increased absolute values of zeta potential have been revealed. Moreover, in 5 individual patients with stage IV GC, expression of MAGE-1 and HER-2/neu mRNA were significantly overexpressed when comparing with healthy donors [8]. All these findings suggested EVs have their own characteristics and functions and EVs should be considered as the target of anticancer therapy. Serum exosomal miRNA panel has been identified as a potential biomarker test for GC. To analysis, the circulating exosomal miRNAs with 20 GC patients and 20 healthy control, four miRNAs (miR-19b-3p, miR-17-5p, miR-30a-5p, and miR-106a-5p) were found involved in GC pathogenesis [9]. Exosomal RNAs derived from human GC cells were characterized by deep sequencing. Exosomes extracting from immortalized normal gastric mucosal epithelial cell line and different GC cell lines have been evaluated. They found the secreted exosomes amount of cancer cell was much higher than normal cell-derived exosomes according to next-generation sequencing technology. On the basis of exosomes microRNA profiles, miR-21 and miR-30a were the most abundant in all types of exosomes [10]. Recently, after comparing the exosomes secreted by both gastric cancer stem-like cells (CSCs) and their differentiated cells, miRNA expression profiles have been identified by Sun et al. miRNA libraries showed that the highly expressed miRNAs were quite different among exosomes from CSCs and differentiated cells according to deep sequencing analysis. Further, 11 significantly differentially expressed miRNAs were identified. 6 miRNAs (miR-1290, miR-1246, miR-628-5p, miR-675-3p, miR-424-5p, miR-590-3p) were up-regulated. The 5 decreased miRNAs were let-7b-5p, miR-224-5p, miR-122-5p, miR-615-3p, miR5787. Among these miRNAs, miR-1290 and miR-1246 were the most abundant in the exosomes from CSCs [11].

#### Tumor-derived EVs affect tumor growth

**Fig. 2:**
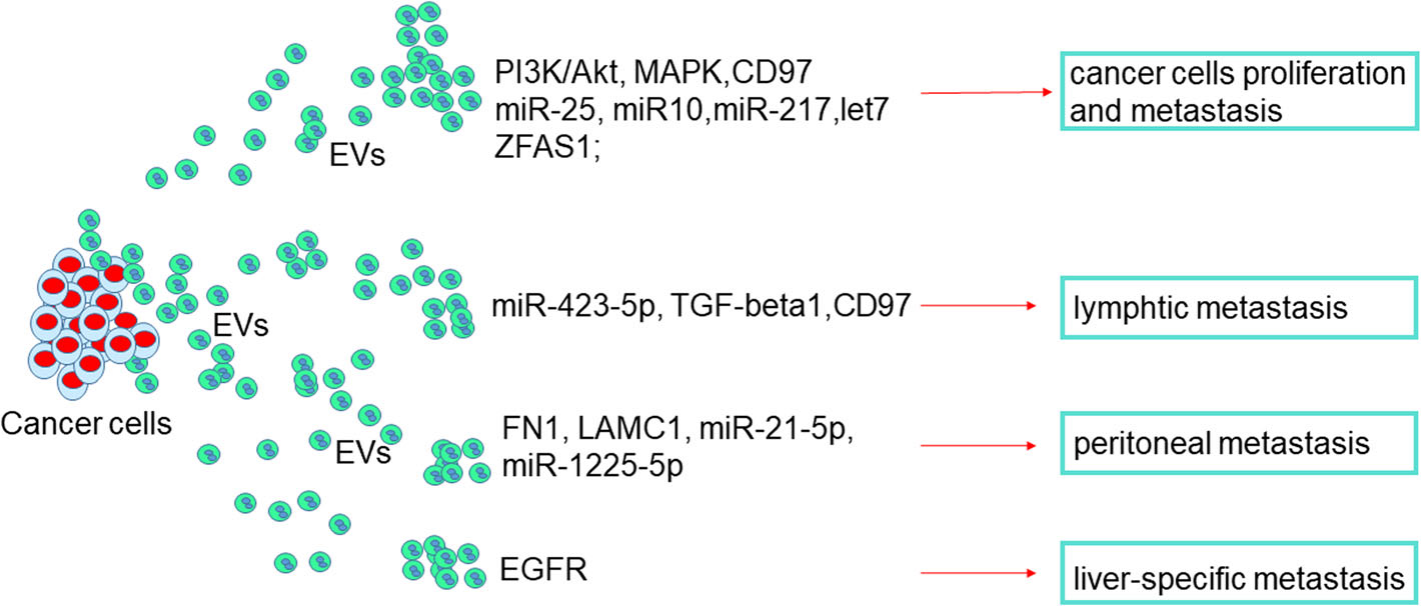
Functions of cancer derived EVs in GC progression and metastasis. The first general mechanism is that GC cells-derived EVs promote tumor cells growth and metastasis through overexpression of multiple proteins, miRNAs and LncRNAs. The second general mechanism is that metastasis, including lymphtic, peritoneal, and liver-specific metastasis, which can be induced by tumor-derived EVs via different pathways in GC. Abbreviations: EGFR, epidermal growth factor receptor

Several proteins and miRNAs that contained in Tumor-derived EVs enhance GC growth have been identified (Fig. 2). CD97 promoted GC cell proliferation and invasion in vitro through exosome-mediated MAPK signaling cascade has been identified by Li et al [12]. SGC-7901 cell derived exosomes mediated the activation of PI3K/Akt and mitogen-activated protein kinase/extracellular-regulated protein kinase pathways, which contributed to enhanced GC cell proliferation [13]. Four potential functional miRNAs in the exosomes were found significantly altered from 67 GC patients’ circular exosomes. Among them, overexpressed exosomal miR-217 and negative associated with CDH1 expression have been identified in GC tissue samples. Moreover, in miR-217 increased cells, the exosomal CDH1 level was reduced, which enhanced cancer cell proliferation and upregulated cell viability [14]. With cultured GC cell lines, let-7 miRNA family was enriched in the extracellular fractions through exosomes to maintain their oncogenesis in a metastatic GC cell line [15, 16]. LncRNA ZFAS1 overexpression has been identified in GC tissues, serum samples, and serum exosomes. Moreover, ZFAS1 could be transferred by exosomes to promote the proliferation and migration of GC cells [17]. Further, cancer cell-derived exosomes on three-dimensional organoids have been reported. They treated gastric organoids (gastroids) with esophageal adenocarcinoma (EAC)-derived EVs and found these EVs could be efficiently taken up by gastroids. Moreover, these EVs promoted gastroids proliferation and cellular viability when comparing to EV-deleted controls. Remarkably, exosome–treated gastroids showed neoplastic morphology than esophageal adenocarcinoma (EAC)-conditioned medium that had been removed of exosomes, which were more compacted and multilayered and contained smaller lumens [18]. Mechanically, these exosomes-induced neoplastic changes in gastroids were the association with the expression of exosomal miRNA, specifically miR-25 and miR-10 [19]. All these findings suggest that exosomal-bearing bioactivators, such as proteins, miRNAs or LncRNAs could be functional signals that among GC cells to induce tumor growth and metastasis.

Some down-regulated proteins or miRNAs in EVs have been studied. LC-MS was used to detect the proteomic profile of the expression of exosomal proteins from the serum of GC patients and healthy control. Serum exosomal TRIM3 was found down-regulated than healthy controls while TRIM3 silence enhanced the progress and metastasis of GC in vitro and in vivo. They also suggested that exosomal TRIM3 may serve as a biomarker for GC diagnosis and the delivery of TRIM3 by exosomes may provide a potential therapy for GC [20]. Gastrokine 1 (GKN1), which plays crucial roles in regulating cell proliferation and differentiation, is another protein that lower expressed in exosomes in GC patients when compared with healthy controls. Importantly, they suggested that human gastric epithelial cells secrete and internalize GKN1 as an exosomal protein to inhibit gastric tumorigenesis [21]. For miR-101, both exosomal and plasma were significantly decreased in GC patients compared with healthy control. Moreover, miR-101 overexpression induced apoptosis by targeting MCL1 and decreased cell migrating and invasion through ZEB1 [22]. The increased knowledge on miRNA greatly promote the progress in clinical implication, where miRNAs could be correlated with prognosis, cancer development, and metastasis.

#### Tumor-derived EVs promote metastasis

The metastasis is an essential event in the development of GC. Lymphatic metastasis is commonly observed in GC. The cancer-related mortality and the communication with tumor microenvironment are the most critical factors in tumor metastasis [23]. EVs play a critical role in remodeling the premetastatic microenvironment (Fig. 2). The concentration of exosomes in serum was significantly higher in GC patients than healthy volunteers. miR-423-5p was remarkedly elevated in the serum exosomes in GC patients and associated with lymph node metastasis. Exosomal miR-423-5p promotes GC growth and metastasis through targeting SUFU and could serve as a marker for GC [24]. After examined the expression of TGF-β1 in the exosomes isolated from the gastroepiploic veins in 61 GC patients and regulatory T (Treg) cells in celiac lymph nodes (LNs). Exosomal TGF-β1 was found significantly associated with lymphatic metastasis and the ratio of Treg cells in lymph nodes of GC. Moreover, exosomes from GC patients could induce Treg cells formation via TGF-β1 [25]. Exosomal CD97 was also suggested to promote GC lymphatic metastasis [26]. Exosomes isolated from an SGC-7901-cell-derived highly lymphatic metastatic cell line (SGC-L) and CD97-knockdown (SGC-L/CD97-KD) cells, and then co-cultured with gastric cancer cells to evaluate the metastatic and lymph node metastasis capacity. Exosomes from the SGC-L cells promoted cell proliferation and invasion as compared with that from SGC-L/CD97-kd cells. Intrafootpad injections of SGC-L exosomes medium actively promoted SGC-L and SGC-L/CD97-kd cell accumulation in the draining lymph nodes and significantly increased CD55, CD44v6, α5β1, CD31, epithelial cell adhesion molecule, and CD151 expression. All these demonstrated the exosome-dependent CD97 plays a central role in premetastatic niche formation in GC [27].

In GC, besides LN metastasis, peritoneal metastasis is a primary metastatic route and common in advanced GC patients. Tumor derived exosomes promoted adhesion to mesothelial cells in GC cells. Internalization of tumor-derived exosomes into mesothelial cells induced the expression of adhesion-related molecules, such as fibronectin 1 (FN1) and laminin gamma 1 (LAMC1). These proteins significantly enhanced adhesion between mesothelial and GC cells [28]. Cancer derived exosomes induced adhesion molecules in mesothelial cells expression, which is essential for the development of peritoneal metastasis of gastric cancer. A critical morphological change in peritoneal metastases is a mesothelial-to-mesenchymal transition (MMT). A monolayer of peritoneal mesothelial cells (PMCs) that lines the peritoneal cavity has been proved to play an important role in this process. Exosomal miR-21-5p induces MMT of PMCs and promotes peritoneal metastasis by targeting SMAD7 has been suggested recently [29]. Exosomal miRNAs in peritoneum lavage fluid could be potential prognostic biomarkers of peritoneal metastasis in GC. Analysis the exosomes isolated from 6 gastric malignant ascites samples, 24 peritoneal lavage fluid samples, and culture supernatants of 2 human GC cell lines, miR-21 and miR-1225-5p were identified as biomarkers in peritoneal recurrence after curative GC resection [30]. GC derived exosomes promote peritoneal metastasis by causing mesothelial barrier destruction and peritoneal fibrosis have been demonstrated [31]. In conclusion, these EVs mediate the peritoneal dissemination in GC by mediating communication between mesothelial cells and cancer cells, to result in the induction of enhancements in tumor growth, migratory, adhesive and invasive abilities, MMT and so on.

Interestingly, EVs play a role in ectopic transfer have been identified. Epidermal growth factor receptor (EGFR-containing exosomes secreted by GC cells can be delivered into the liver and were ingested by liver stromal cells. The transferred EGFR is proved to inhibit miR-26a/b expression an activate hepatocyte growth factor (HGF). Then, the upregulated paracrine HGF binds the c-MET receptor on the migrated cancer cells to facilitate the seeding and proliferation of metastatic cancer cells. Thus, EGFR-containing exosomes could favor the progress of a liver-like microenvironment promoting liver-specific metastasis [32].

#### EVs and biomarkers

Recently, some exosomal proteins, miRNAs, and LncRNAs are up-regulated in the serum of GC patients, which showed that these EVs might be diagnostic markers for GC. Due to their located in body fluids, EVs-based diagnostic is suggested to be optimal candidates for noninvasive diagnosis. In 30 gastric juice-derived exosomes, BarH-like 2 homeobox protein (BARHL2) showed high levels of methylation. Interestingly, BARHL2 methylation generated an area under the curve of 0.923 with 90% sensitivity and 100% specificity concerning recognizing GC patients from healthy controls when analysis of gastric juice-derived exosome DNA samples [33]. All these results suggested that methylation analysis of BARHL2 using gastric juice-secreted exosome DNA could be beneficial for early diagnosis of GC in clinical settings. As the same for early-stage GC, tumor-originated exosomal IncUEGC1 is another promising highly sensitive, stable, and non-invasive biomarkers. After comparing RNA-sequencing analysis of plasma exosomes between five healthy individuals and 10 stage І GC patients, lncUEGC1 and lncUEGC2 were confirmed to be remarkably up-regulated in exosomes derived from early GC patients [34]. Plasma long noncoding RNA LINC00152 encompassed by exosomes is a potential stable biomarker for GC. There are no differences between the levels of LINC00152 in plasma and exosomes. All these results suggested that one of the possible mechanisms of LINC00152 can be detected in plasma in stable existence in blood was because it is protected by exosomes [35]. Therefore, exosomes can be applied in gastric cancer diagnosis as a novel blood-based biomarker. Serum exosomal long noncoding RNA HOTTIP was significantly higher in 126 GC patients than in 120 normal control people, which suggested that HOTTIP is a potential novel diagnostic and prognostic biomarker test for GC [36]. Moreover, plasma exosomal miR-23b could be a liquid biomarker for prediction of recurrence and progression of GC patients in each tumor stage [37].

## Roles of tumor-derived EVs in GC microenvironment

**Fig. 3:**
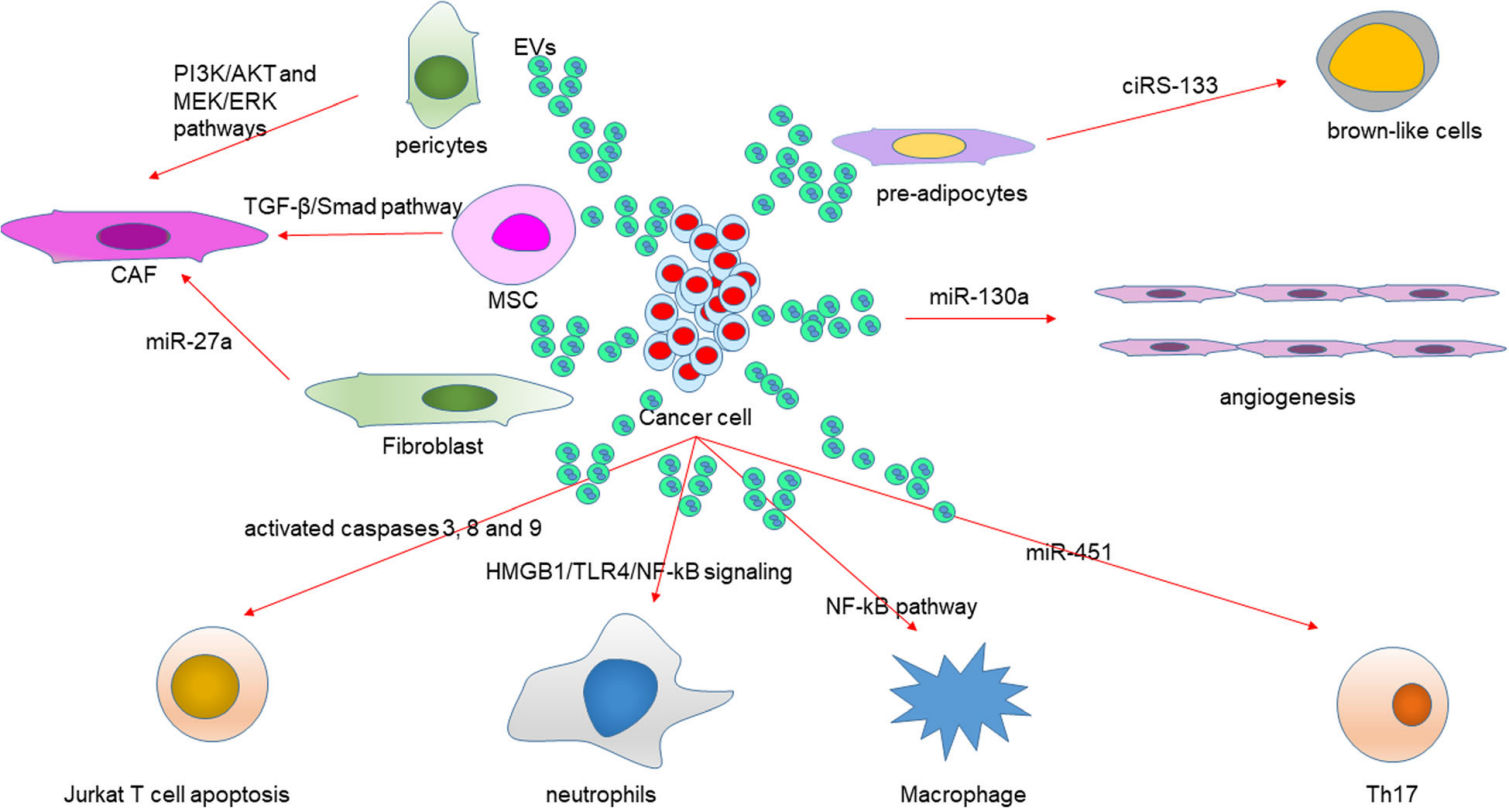
The functional network of cancer derived EVs in GC microenvironment. GC cells-derived EVs promote angiogenesis via releasing miR-130a. Pericytes, MSCs, and fibroblasts absorbed EVs to induce CAFs transformation in tumor microenvironment through different pathway or miRNAs in cells. The functions of cancer cells-derived EVs in adipocytes differentiation. Different immune cells in tumor microenvironment can be affected by tumor-derived EVs. GC-derived EVs inhibit T cell immunity, polarize neutrophils to a pro-tumor phenotype, induce macrophages to release more proinflammatory factors and active Th17 to promote cancer progression. Abbreviations: GC, gastric cancer; MSC, mesenchymal stem cell; CAF, cancer-associated fibroblast

In this part, we will focus on the effects of EVs on the tumor microenvironment. As a carrier, EVs play a vital role in the communication between tumor cells and tumor microenvironment (Fig. 3). Tumor microenvironment contains complex components, such as extracellular matrix (ECM), immune cells, stromal cell, endothelial cell, blood vessels, non-epithelial cells such as fibroblasts. In exosome, the most expression proteins belong to the *tetraspanins* family, such as CD63, which is the marker of isolated exosomes [38]. Recently, a study clarified the relationship between CD63 expression in stromal cells and GC cells and clinical-pathologic factors with 595 GC patients. They found CD63 was mainly expressed on the cell membranes of cancer cells, and in the cytoplasm of stromal cells. The 5-year survival rate was negatively correlated with CD63 expression. Theas results suggested CD63 might be a prognostic marker and CD63-positive exosomes might be the interaction between GC cells and stromal cells [39]. Therefore, cancer-derived exosomes play a critical role in the establishment of the tumor microenvironment.

### The effects of tumor-derived EVs in the angiogenesis

miR-130a is involved in angiogenesis, exosome-derived miR-130a activates angiogenesis in GC through interacting C-MYB in vascular endothelial cells (Fig. 3). Exosomes in GC cells delivered miR-130a into vascular cells to enhance angiogenesis and tumor develop through binding *c-MYB* both in vitro and in vivo [40]. After treated with exosomes released from GC cell lines after irradiation, the proliferation, migration and invasion capacity of Human Umbilical Vein Endothelial Cells (HUVEC) are induced. Importantly, the increased progression of these HUVEC is counteracted by the VEGFR-2 inhibitor Apatinib. Therefore, bonding ionizing radiation and VEGFR inhibitors is a potentially valid treatment in GC [41]. Cell-derived EVs mediate the delivery of miR-29a/c to suppress angiogenesis in gastric carcinoma. miR-29a/c decreases VEGF expression and releases in GC cells, inhibiting the growth of vascular cells. Moreover, in a tumor implantation mouse model, released MVs with overexpressed miR-29a/c in significantly inhibited the growing rate of the tumors and vasculature in vivo. These results suggested a novel anti-cancer strategy with miR-29a/c containing MVs to block angiogenesis to decrease tumor growth [42].

### The effects of tumor-derived EVs in fibroblasts

In the tumor microenvironment, cancer-associated fibroblasts (CAFs) are necessary for cancer progression (Fig. 3). There are three main classes of CAFs: mesenchymal stem cells (MSCs), epithelial-to-mesenchymal (EMT) transition cells, and tissue-resident cells. Wang et al. found that exosomal miR-27a derived from GC cell regulates the transformation of fibroblasts into CAFs [43]. They found miR-27a in exosomes was highly expressed in GC cell lines. miR-27a reprogrammed the fibroblasts into CAFs and promoted the cancer development. Apart from fibroblast transformed to CAFs, cancer cell-derived exosomes are also involved in regulating the transition of pericytes to CAFs. Exosomes released by gastric cancer cells promoted pericytes proliferation and migration and induced the expression of CAFs marker in pericytes has been identified. They also identified that tumor-derived exosomes activated the PI3K/AKT and MEK/ERK pathways, and inhibited BMP pathway to reverses cancer exosomes-induced CAFs transition [44]. Moreover, cancer cell-derived exosomes regulated the differentiation of human umbilical cord-derived MSCs (hucMSCs) to CAFs have been revealed. TGF-β transfer and TGF-β/Smad pathway activation were mediated by exosomes to trigger the differentiation of hucMSCs to CAFs [45].

### The effects of tumor-derived EVs in immune cells

Tumor-derived EVs contain molecular that can promote immune cell dysfunction and transform the microenvironment suitable for their growth and metastasis (Fig. 3). Tumor-derived exosomes can inhibit T cell immunity and direct immune cells to promote tumor progression [46]. GC cells derived exosomes activated NF-κB pathway to induce macrophages to release more proinflammatory factors, resulting in promoted cancer cell proliferation, migration, and invasion. These results exhibited the function of exosomes in eliciting macrophage activation to promote GC progression [47]. The tumor could polarize neutrophils to a pro-tumor phenotype. Zhang et al suggested that GC cell-derived exosomes prolonged neutrophils survival and induced inflammation factor expression in neutrophils. Then, GC cell migration could be promoted by these GC cell-derived exosomes activated neutrophils. Furthermore, they demonstrated that autophagy and pro-tumor activation of neutrophils through HMGB1/TLR4/NF-kB signaling were induced by GC cell-derived exosomes [48]. Exosome-encompassed miR-451 from cancer cells could increase the differentiation of T-helper 17 (TH17) cells in low glucose condition. Exosomal miR-451 could be an indicator for poor prognosis of post-operation GC patients and related to increased Th17 distribution in GC by promoting mTOR signaling pathway activity. These results enhance our study of how tumor cells modify the microenvironment through exosomes [49]. GC-derived exosomes activated caspases 3, 8 and 9 to induce Jurkat T cell apoptosis has been identified [50]. GC-derived exosomes effectively educated monocytes to differentiate into PD1^+^ TAMs with M2 phenotypic and functional characteristics. CD8^+^ T-cell function was suppressed by PD1^+^ TAMs and this immunosuppressive activity can effectively be enhanced through inducing PD1 signal. Therefore, GC-derived exosomes can effectively induce PD1^+^ TAM generation that creates conditions that promote GC progression [51].

### The effects of tumor-derived EVs in white adipose browning

Cancer-related cachexia is a metabolic syndrome in cancer and circRNAs in plasma exosomes are involved in white adipose tissue (WAT) browning and play a critical role in cancer-associated cachexia (Fig. 3). GC cells derived exosomes transfer ciRS-133 into pre-adipocytes, accelerating the differentiation of pre-adipocytes into brown-like cells by activating PRDM16 and suppressing miR-133 [52].

## Roles of microenvironment-derived EVs in GC

**Fig. 4:**
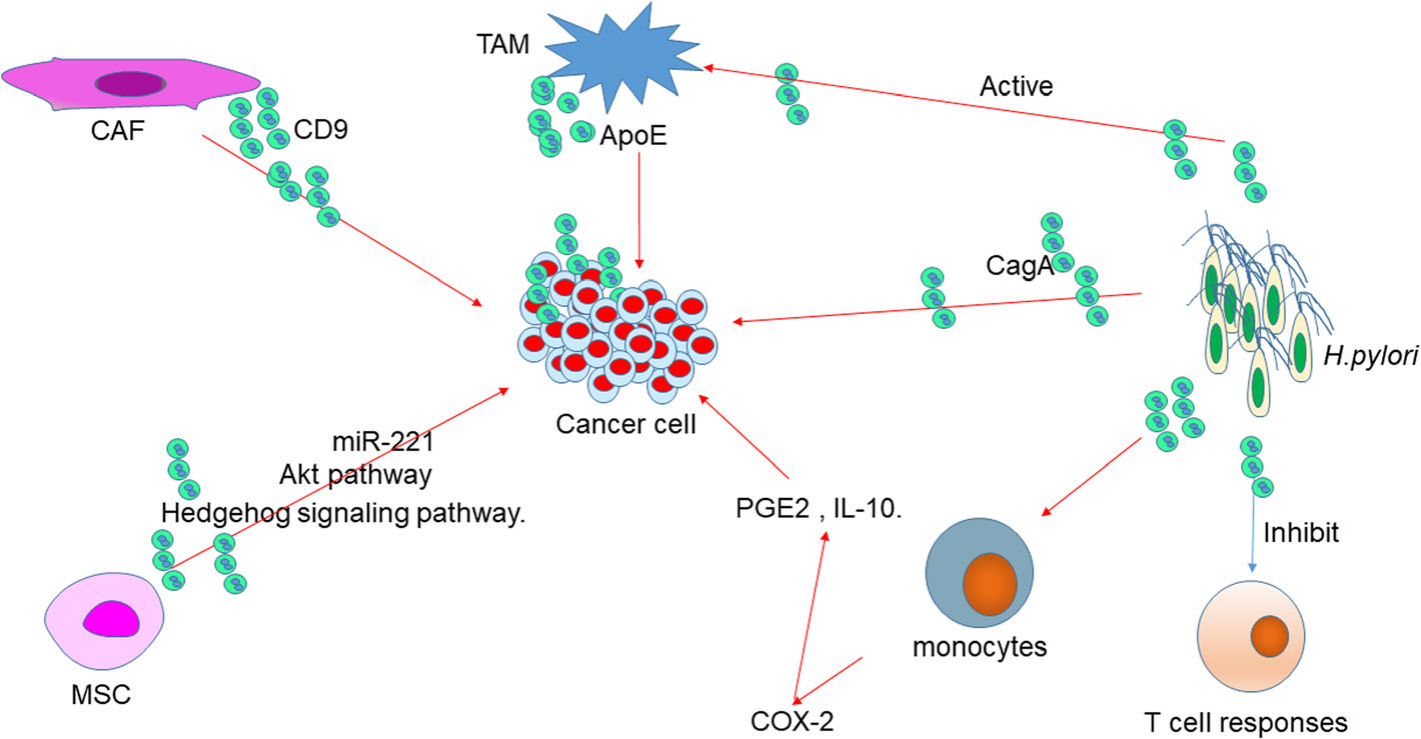
The regulation network of microenvironment-derived EVs as well as *H.pylori*-derived EVs in GC. EVs secreted by CAF, MSC, and TAM induce GC progression through different pathways and molecules. *H.pylori* releases CagA-containing EVs and other EVs that inhibit T cell responses, active monocytes to induce COX-2 expression, and active TAM to induce gastric carcinogenesis. Abbreviations: TAM, tumor-associated macrophage; CAF, cancer-associated fibroblast; MSC, mesenchymal stem cell

Exosomes derived from cancer cells played a critical role in intracellular communications. Similarly, the effect of exosomes from tumor microenvironment on the progression of GC cells is also important (Fig. 4). Exosomes from CAFs significantly stimulated the migration and invasion of scirrhous-type gastric cancer cells. CD9-positive exosomes from CAFs activate the migration ability of scirrhous-type GC cells [53].

TAMs are the major component in the tumor microenvironment. In GC, M2 phenotype is the primary macrophage subpopulation. M2 exosomes enhanced migration of GC both in vitro and in vivo has been identified. The mechanism has been proved. An intercellular transfer of ApoE-activating PI3K-Akt signaling pathway in recipient GC cells to influence the cytoskeleton-supporting migration was mediated by M2 macrophage-derived exosomes. These results suggested that transfer of functional ApoE protein from TAMs to the tumor cells promotes the migration of gastric cancer cells were mediated by the exosome [54].

MSCs are a component of the tumor microenvironment. Exosomes released by MSCs can deliver bioactive molecules, including proteins and nucleic acid, to other cells in the tumor environment to affect the progression of the tumor. Firstly, Gu et al found MSC-derived exosome promoted GC growth in vivo and stimulated CAF differentiation of MSCs [45, 55]. Then they found exosomes derived from human MSCs enhanced GC malignant properties and induced the EMT and cancer stemness in GC cells through the activation of the Akt pathway [56]. GC cell growth was promoted by human bone marrow MSC (hBMSCs)-derived exosomes through the activation of the Hedgehog signaling cascade. Moreover, suppression of Hedgehog signaling cascade significantly inhibited the process of hBMSC-derived exosomes on tumor growth [57]. The state of p53 in MSCs to impact the bioactive molecule secretion of exosomes to promote cancer progression has been revealed. The exosome concentration was significantly higher in p53^−/−^ mouse bone marrow MSC (mBMMSC) than that in p53 wild type mBMMSC (p53 ^+ / +^ mBMMSC). Moreover, P53^−/−^mBMMSC exosomes containing abundant UBR2 could be internalized into p53 ^+ / +^ mBMMSC and murine foregastric carcinoma cells and cause the upregulation of UBR2 in these cells which enhanced cell proliferation, migration, and the expression of stemness related genes. Finally, they indicated that p53^−/−^mBMMSC exosomes could deliver UBR2 via regulating the Wnt/β-catenin pathway to target cells and promote gastric cancer growth and metastasis [58]. The poor clinical prognosis of GC was positively associated with high expression of miR-221 in exosomes in the peripheral blood. Transfected miR-221 oligonucleotides to bone marrow mesenchymal stem cells (BM-MSCs), then exosomes were extracted. These EVs serve as high-efficiency nanocarriers, which can provide sufficient miR-221 oligonucleotides to effectively reprogramme the tumor microenvironment and tumor aggressiveness [59].

## Roles of *H. pylori* derived EVs in GC

*H.pylori* is an important factor in GC and triggers chronic inflammation. The role of *H.pylori* -derived EVs have been identified (Fig. 4). CagA (Cytotoxin-associated gene A) is a major virulence factor in *H.pylori*. In gastric juices from GC patients, *H. pylori*-derived EVs were upregulated when compared with healthy controls. Stomach epithelial cells selectively targeting and taken up *H. pylori* -derived EVs. *H. pylori*-derived EVs enhanced in the gastric juices of gastric adenocarcinoma patients and promoted inflammation mainly via specific targeting of gastric epithelial cells [60]. CagA was present in serum-derived exosomes in patients infected with cagA-positive *H. pylori* has been reported. These exosomes may from gastric epithelial cells which inducibly expressing CagA secret exosomes, and then entered into circulation, transferring CagA to distant organs and tissues [61]. Pan et al found association between *H.pylori*-infected GC cells and macrophages through exosome. They also demonstrated that *H.pylori*-induced exosomal MET educated tumor-associated macrophages to promote gastric cancer progression [62]. Human T cell responses was inhibited by *H.pylori* outer membrane vesicles via induction of monocyte cyclo-oxygenase-2 (COX-2) expression has been proved. The outer membrane of *H. pylori* releases vesicles to modulate the immune system. Subsequent T cell proliferation was inhibited by PBMC significantly after addition of *H. pylori* outer membrane vesicles in a COX-2 dependent manner. Expression of COX-2 was significantly induced by *H. pylori* outer membrane vesicles which was inducing by the monocytes present and significantly increased levels of PGE2 and IL-10. These results suggest that *H. pylori* outer membrane vesicles can suppress human T cell responses is not only through a direct effect on the T cells but also results from the induction of COX-2 expression in monocytes [63].

## Roles of EVs in GC drug resistance

The poor prognosis of GC is due to multiple factors, including resistance to conventional therapies. Paclitaxel is a first-line chemotherapeutic drug for GC. Recently, paclitaxel-resistant gastric cancer cell line (MGC-803R) cell-derived exosomes could be efficiently taken up by paclitaxel-sensitive MGC-803 (MGC-803S) cells has been observed. Subsequently, miR-155-5p was proved highly expressed in MGC-803R-exosomes and could be transferred into MGC-803S cells to induce its chemoresistance phenotypes. Furthermore, exosomal miR-155-5p directly inhibiting GATA binding protein 3 (GATA3) and tumor protein p53-inducible nuclear protein 1 (TP53INP1) to induce chemoresistant phenotypes from paclitaxel-resistant GC cells to the sensitive cells have been proved [64]. MSCs are also implicated in the drug-resistance in GC. Exosomes derived from human MSCs could afford drug resistance to 5-fluorouracil in GC cells both in vitro and in vivo*,* which was correlated with elevated MDR-associated MDR, MRP, and lung resistance protein mRNA and protein levels, and a decrease in apoptotic rate. Further, the mechanism of MSC-exosomes triggered drug resistance in GC cells was the activation of calcium/calmodulin-dependent protein kinases and Raf/MEK/ERK kinase cascade have been found [65]. Exosomes secreted by tumor-associated macrophages (TAMs) mediated cisplatin resistance in GC has been identified. This project of drug-resistance was supported by in vivo studies. MFC cells, which was treated with or without EVs derived from TAM-like macrophages, was subjected to a subcutaneous model. Then administrated with cisplatin for 10 days. The presence of the EVs had minimal effect on tumor growth, however they substantially inhibited the anti-cancer effects of cisplatin. With miRNA microarray analysis, miR-21a-5p in exosomes from M2 polarized macrophage was the most abundant miRNAs. Exosomal miR-21 can be directly transferred from macrophages to GC cells to confer the chemotherapy resistance in cancer cells, inhibit cell apoptosis and activation PI3K/AKT pathway by regulating PTEN [66]. These findings reveal the profound effects of EVs, both cancer-derived or environment-derived EVs on modifying GC cells in the development of drug resistance.

## Roles of EVs in the GC treatment

Furthermore, EVs are potential natural carriers of anticancer agents, which suggested that exosome-based treatment of GC may be an effective approach. Macrophages derived exosomes transfer exogenous miR-21 inhibitor into BGC-823 GC cells to regulate its proliferation. Furthermore, when comparing to conventional transfection methods, exosome mediated-miR-21 inhibitor transfer resulted in functionally less cellular toxicity and more efficient inhibition [67]. These results contribute to our understanding of the functions exosomes as a carrier for therapy of GC. Exosomes serve as nanoparticles to transfer anti-miR-214 to reverse chemoresistance to Cisplatin in GC have been identified [68]. Hepatocyte growth factor (HGF) siRNA packed in exosomes can be transported into GC cells, where it suppressed proliferation and migration of both cancer cells and vascular cells. Moreover, in vivo, exosomes were also able to deliver HGF siRNA, inhibiting the growth rates of tumors and blood vessels. These results suggested that exosomes by delivering HGF siRNA could be served as nanoparticles to suppress tumor growth and angiogenesis in GC [69]. The role of exosomes as a novel type of cancer vaccine has been studied. Higher concentrations of heat shock proteins, Hsp70 and Hsp60 were found in exosomes from heat-treated malignant ascites of gastric cancer patients than exosomes derived from untreated malignant ascites obtained from GC patients. In vitro studies suggested that exosomes derived from heat-treated malignant ascites can promote a tumor-specific cytotoxic T lymphocyte (CTL) response and induce dendritic cell maturation. These results suggested that exposure to heat stress could accelerate the immunogenicity of exosomes obtained from malignant ascites of GC patients [70]. High dose of a proton-pump inhibitor (PPIs) inhibited the release of exosomes, which packed miRNAs to regulate the tumor malignancy and microenvironment [71]. Trastuzumab emtansine (T-DM1) carries a cytotoxic drug (DM1) to HER2-positive cancer through an antibody-drug conjugation method. Cancer-derived exosomes also contained the target of T-DM1 (HER2). Therefore, exosome-bound T-DM1 whether contributing to the activity of T-DM1 has been studied. Exosomes derived from HER2-positive cancer cells associated with T-DM1, and T-DM1 may be carried to other cancer cells via exosomes leading to decrease viability of the recipient cells. Therefore, trastuzumab-emtansine was carried by cancer-derived exosomes from HER2-positive cancer cells into cancer cells leading to growth suppression and caspase activation [72].

## Conclusions and future directions

Circulating tumor cells, circulating tumor DNA, tumor exosomes, and microRNAs, are involved in liquid biopsies. Among them, increasing attention is being paid to EVs. The advantage of EVs relies on their ubiquitous presence, their particular DNA /RNA/ protein profile, and their most efficient transfer in target cells. Identify these genomic profiling has the potential to assess various biomarkers for early detection of GC. Moreover, study EVs in GC also provide appropriate therapy and provide monitor to the effect of therapy. On the other hand, although these studies have prompted the clinical applications of EVs, many problems need to be further elucidated. Firstly, more accurate and standardized purification methods are required for the clinical samples. Secondary, there are multiple bioactivators in EVs and what is the main functional components in EVs. Thirdly, although RNAs have been the focus of EVs in GC for the last decade, and which component may the most suitable for biomarkers identification? The basic mechanisms/characteristics of EVs biology in GC have yet to be determined. Therefore, continued in-depth investigation is required. In summary, the deep understanding of EVs will provide better clinical translational potential for GC.

## Abbreviations

BARHL2: BarH-like 2 homeobox protein
BMMSC: Bone marrow MSC
CAFs: Cancer-associated fibroblasts
CagA: Cytotoxin-associated gene A
CIN: Chromosomal instability
COX-2: Cyclo-oxygenase-2
CSCs: Cancer stem-like cells
CTL: Cytotoxic T lymphocyte
EAC: Esophageal adenocarcinoma
EBV: Epstein-Barr virus
ECM: Extracellular matrix
EGFR: Epidermal growth factor receptor
EMT: Epithelial-to-mesenchymal
EVs: Extracellular vesicles
FN1: Fibronectin 1
gastroids: Gastric organoids
GATA3: GATA binding protein 3
GC: Gastric cancer
GKN1: Gastrokine 1
GS: Genomically stable tumors
*H. pylori*: Helicobacter pylori
HGF: Hepatocyte growth factor
HGF: Hepatocyte growth factor
hucMSCs: Human umbilical cord-derived MSCs
HUVEC: Human Umbilical Vein Endothelial Cells
LAMC1: Laminin gamma 1
LNs: Lymph nodes
MGC-803R: Paclitaxel-resistant gastric cancer cell line
MGC-803S: Paclitaxel-sensitive MGC-803
miRNA: microRNAs
MMT: Mesothelial-to-mesenchymal transition
MSCs: Mesenchymal stem cells
MSI: Microsatellite unstable tumors
MVs: Microvesicles
PMCs: Peritoneal mesothelial cells
PPIs: Proton-pump inhibitor
SGC-L: SGC-7901-cell-derived highly lymphatic metastatic cell line
SGC-L/CD97-KD: CD97-knockdown
TAMs: Tumor-associated macrophages
TCGA: The Cancer Genome Atlas
TH17: T-helper 17
TP53INP1: Tumor protein p53-inducible nuclear protein 1
Treg: Regulatory T
WAT: White adipose tissue
